# ChIP-seq identifies *McSLC35E2* as a novel target gene of *Mc*Nrf2 in *Mytilus coruscus*, highlighting its role in the regulation of oxidative stress response in marine mollusks

**DOI:** 10.3389/fphys.2023.1282900

**Published:** 2023-10-06

**Authors:** Longmei Qiu, Xinglu Chen, Li Zhu, Ronghui Yao, Pengzhi Qi

**Affiliations:** National Engineering Research Center of Marine Facilities Aquaculture, Marine Science and Technology College, Zhejiang Ocean University, Zhoushan, Zhejiang, China

**Keywords:** marine mussels, Nrf2, ChIP-seq, SLC35E2, oxidative stress

## Abstract

NF-E2-related factor 2 (Nrf2) plays a crucial role in the oxidative regulatory process, which could trigger hundreds of antioxidant elements to confront xenobiotics. In the previous study, we identified Nrf2 from the marine mussel *Mytilus coruscus*, and the findings demonstrated that *Mc*Nrf2 effectively protected the mussels against oxidative stress induced by benzopyrene (Bap). In order to delve deeper into the underlying mechanism, we utilized Chromatin Immunoprecipitation followed by sequencing (ChIP-seq) technology to systematically identify potential novel target genes of *Mc*Nrf2. A total of 3,465 potential target genes were screened, of which 219 owned binding sites located within the promoter region. During subsequent experimental verification, it was found that *Mc*SLC35E2, a candidate target gene of *Mc*Nrf2, exhibited negative regulation by *Mc*Nrf2, as confirmed through dual luciferase and qRT-PCR detection. Further, the enzyme activity tests demonstrated that *Mc*Nrf2 could counteract Bap induced oxidative stress by inhibiting *Mc*SLC35E2. The current study provides valuable insights into the application of ChIP-seq technology in the research of marine mollusks, advancing our understanding of the key role of Nrf2 in antioxidant defense mechanisms, and highlighting the significance of SLC35E2 in the highly sophisticated regulation of oxidative stress response in marine invertebrates.

## 1 Introduction

In recent years, the thick shell mussel *Mytilus coruscus* has gradually developed into a model organism for studying marine invertebrates responses to environmental changes, including natural influences such as temperature rise and acidification, as well as environmental pollution from organic and inorganic substances (Zhao et al., 2020; Dong et al., 2023; Wang et al., 2023). Our research focuses on the molecular-level responses of *M. coruscus* to polycyclic aromatic hydrocarbons (PAHs) pollution, particularly on its member benzo(a)pyrene (Bap). Bap has been proven to cause severe harm to marine organisms, including immune system disruption, metabolic inhibition, induction of mutagenic reactions, and tissue damage (Xiu et al., 2014; Kim et al., 2017). To adapt and resist stress, the cellular organisms activate a multi-layered defense system that is closely associated with various cellular processes, with transcription regulation being one of the most crucial components of this integrated system (Hirotsu et al., 2012). NF-E2-related factor 2 (Nrf2), identified as a fundamental leucine zipper nuclear transcription factor, holds a central position in cellular reactions to diverse environmental contaminants (Shaw et al., 2020). The primary function of Nrf2 involves overseeing the expression of numerous antioxidant genes, consequently enabling the activation of the Nrf2 signaling pathway to proficiently govern cellular antioxidant and detoxification reactions (Liu et al., 2021). Nrf2 is also believed to be involved in host defense during the antimicrobial immune response (Wang et al., 2021c). Furthermore, there is increasing evidence suggesting that Nrf2 exerts significant effects on lipid, carbohydrate, and amino acid metabolism (Hayes and Dinkova-Kostova, 2014). These characteristics contributes to its ability to efficiently coordinate different forms of stress responses (Zago et al., 2021). In our previous study, *Mc*Nrf2 was identified from *M*. *corus*cus, and the experimental results unequivocally demonstrated that *Mc*Nrf2 efficiently plays a pivotal role in protecting the mussels from oxidative stress induced by Bap (Qi and Tang, 2020). Thereafter, the transcriptional regulation mechanism of *Mc*Nrf2 against Bap oxidation is the focus of our next research.

Understanding transcriptional regulation is essential to comprehending the gene regulatory networks behind various cellular pathways and processes. Accurate mapping of transcription factor binding sites (TFBS) on a genome-wide scale can provide invaluable insights into gene regulation. Protein-DNA interactions are key to this mapping process and an extensive genome-wide map of interaction data is necessary to build meaningful models of TFBS (Farnham, 2009). Chromatin immunoprecipitation (ChIP) is a widely used technique to investigate the mechanisms of protein-DNA binding in living cells. This technique uses antibodies to isolate specific proteins or nucleosomes, thereby enriching for DNA fragments bound to them. ChIP is a powerful tool for probing protein-DNA interactions as it allows to accurately pinpoint gene regulatory regions and quantify their respective activities (Solomon et al., 1988). NGS (next-generation sequencing) has rapidly revolutionized the landscape of available genomic assays, transforming them into powerful and versatile tools (Shendure and Ji, 2008). Chromatin immunoprecipitation followed by sequencing (ChIP–seq) was one of the typical applications of NGS. In ChIP-seq, the DNA segments of interest are sequenced directly, rather than hybridized on an array, thus providing greater coverage, higher resolution, and greater dynamic range, ultimately producing better data (Park, 2009). Johnson et al. (2007) demonstrated that ChIP-seq could improves the sensitivity and specificity of genome-wide localization of transcription factor binding sites. Despite the extensive and mature application of ChIP-seq in higher organisms such as human beings, its application in lower eukaryotes is still very rare. Thus far, only a few research groups have made attempts to incorporate this technology into studies involving marine mollusks. Li et al. (2022) employed ChIP-seq to analyse the genes regulated by Heat shock transcription factor 1 (HSF1) in the Pacific oyster *Crassostrea gigas*, and found a number of Heat shock protein (HSP) genes bind to HSF1. This research unveiling the application of ChIP-seq technology in marine mollusks.

In the present study, we employed ChIP-seq assay to comprehensively screen for potential novel target genes of *Mc*Nrf2, followed by subsequent experimental validation. We revealed for the first time that solute carrier family 35 member E2 (SLC35E2) functions as a target gene for *Mc*Nrf2, which is demonstrated by the binding of *Mc*Nrf2 to the promoter region of *Mc*SLC35E2. Subsequent dual-luciferase and qRT-PCR assays further confirmed this fact. Further, the enzyme activity tests determined that *Mc*Nrf2 could target *Mc*SLC35E2 to antagonize Bap induced oxidative stress. The current study provides valuable insights into the application of ChIP-seq technology in the research of marine mollusks. Moreover, the research findings have advanced our understanding of the key role of Nrf2 in antioxidant defense mechanisms and highlights the significance of SLC35E2 in the highly sophisticated regulation of oxidative stress response in marine invertebrates.

## 2 Materials and methods

### 2.1 Experimental materials

A total of 200 healthy *M. coruscus* mussels were obtained from Donghe Market, Zhoushan City, Zhejiang Province. These mussels were acclimated in a tank at a temperature of 20°C for 1 week. The seawater used had a salinity of 30% ± 1% and a pH of 8.0 ± 0.3. The seawater was renewed every 2 days, and the mussels were fed with Spirulina powder on a daily basis.

### 2.2 ChIP sample preparation

The digestive gland cells of mussels were extracted and 20 mL formaldehyde fixative was added to make the final concentration 1%. After incubation at room temperature on a 100 × *g* for 10 min, the cells were added 10 mL of glycine termination solution with a 5 min centrifugation at 300 × *g*, 4°C. The cells were then washed twice with phosphate buffer containing 1 mM PMSF to remove any remaining formaldehyde. After washing, 1 mL of lysis buffer was added, lysed on ice for 30 min, followed by a cells collection by centrifugation at 5,000 × *g*, 4°C for 10 min. Next, 350 μL of pre-warmed digestion buffer was added, and the mixture was incubated at 37°C for 5 min. The cut chromatin was separated, and 10 μL of input DNA was labeled and kept as a control for ChIP samples. The ChIP reaction system was prepared and then incubated overnight at 4°C on a rotating shaker. Subsequently, the magnetic beads were washed, and the chromatin was eluted. Uncross linking and proteinase K treatment were performed afterward. Finally, the resulting DNA was purified, and the detailed steps were described in the Magnetic Chromatin Immunoprecipitation Kit (Active Motif, CA, United States).

### 2.3 Illumina sequencing

ChIP-seq libraries were generated following the Illumina ChIP-seq library construction protocol. The chip DNA was fragmented into fragments of approximately 200 bp in length. These DNA fragments then underwent end repair and A-tailing processes. Subsequently, adaptor ligation was performed to attach sequencing adaptors to the DNA fragments. To ensure high-quality libraries, the quality assessment of DNA library products was conducted using the Agilent 2200 TapeStation (Agilent Technologies, United States) and Qubit (Thermo Fisher Scientific, United States). Subsequently, the libraries were subjected to pair-end 150 bp sequencing on the Illumina platform (Illumina, NovaSeq 6000, United States) at Ribobio Co., Ltd. (Ribobio, China).

### 2.4 ChIP-seq data analyses

The raw fastq sequences were processed using Trimmomatic tools (v0.36) with the following options: TRAILING: 20, SLIDINGWINDOW: 4:15, MINLEN: 52. This process was performed to eliminate trailing sequences with a phred quality score below 20 and to obtain uniform sequence lengths for subsequent clustering procedures (Bolger et al., 2014). The genome alignment, based on the UCSC Genome Browser version, was conducted using bowtie2 (version: 2.5.1) to obtain unique mapping reads, aligning them to the *M. coruscus* genome (unpublished) (Langmead and Salzberg, 2012). Subsequently, MACS3 (version 3.0.0a7) was utilized for peak calling, with the corresponding input sample serving as the control for the analysis (Zhang et al., 2008). Then using Hommer (version:4.11.1) to annotate the peaks. The nucleotides in peaks region were used for detection of the consensus m6A motif by DREME (version: 5.5.1) and MEME (version: 5.5.1) (Heinz et al., 2010). Motif central enrichment was performed by CentriMo (version: 5.5.1) (Ma et al., 2014). Kyoto Encyclopedia of Genes and Genomes (KEGG) pathway enrichment analysis was performed using KOBAS3.0/ the “clusterProfiler” package in R Bioconductor. The enriched results were restricted to KEGG pathway terms. The KEGG pathway terms with adjusted *p* < 0.05 were considered to be significant.

### 2.5 Validation of target genes by a dual luciferase assay

To validate the relationship between *Mc*Nrf2 with a candidate target gene *McSLC35E2*, dual luciferase assays were performed using the Dual-Glo^®^ Luciferase Assay System (Promega, Madison, WI, United States). The experimental procedure followed the instructions provided by the manufacturer. The ChIP-seq data revealed the enrichment of the potential binding site, i.e., the region of the SLC35E2 promoter where Nrf2 is capable of binding. The region approximately 1 kb upstream of the *SLC35E2* gene was cloned into the pGL3-control luciferase reporter plasmid. Additionally, the *Nrf2* fragment was cloned for insertion into the pcDNA3.1 plasmid. After cloning, the recombinant plasmids were transfected into the recipient cells, and subsequent extraction was carried out for sequencing verification. Plasmids that underwent successful sequencing were co-transfected into cells. Then, the activity of the reporter gene was assessed using the Dual-Luciferase Reporter Assay System. The fluorescence signals for both Firefly and Renilla luciferase were captured using the Varioskan Flash Multimode Reader from Thermo Fisher Scientific (Waltham, MA, United States). The recorded fluorescence values for each experimental group were then utilized to evaluate the regulatory effect of Nrf2.

### 2.6 Determination of expression patterns by qRT-PCR

After 1 week of individual domestication, SFN (Sulforaphane), ML385 (N-[4-[2,3-Dihydro-1-(2-methylbenzoyl)-1H-indol-5-yl]-5-methyl-2-thiazolyl]-1,3-benzodioxole-5-acetamide), and PBS were administered via injection. Digestive gland tissues of *M. coruscus* were collected at 24 h. Three individuals were selected from each group for sampling. Total RNA was extracted using the RNA extraction kit from Solarbio (Beijing, China), followed by a reverse transcription using cDNA synthesis kit (Solarbio, Beijing, China). The housekeeping gene β-actin was employed as a control gene in our study. The qRT-PCR was conducted utilizing the SYBR Green Real-Time PCR Mix (Takara, Nanjing, China) on a ABI 7500 Fast Real-Time PCR System (Applied Biosystems, Foster, CA, United States) and. Data analysis was carried out using the 2^−ΔΔCT^ method (Livak and Schmittgen, 2001). The primers were listed in Supplementary Table S1.

### 2.7 ROS and T-AOC determination

The eukaryotic expression recombinant plasmids of *Mc*Nrf2 and *Mc*SLC35E2, which were prepared in our laboratory, were collected and their final concentration was diluted to 300 ng/μL. *M. coruscus* individuals (Net weight: 19.8 ± 0.3 g) were randomly divided into 5 groups, each consisting of 6 mussels. The adductor muscle of each mussel was injected with either 100 µL *Mc*Nrf2, *Mc*SLC35E2, or 200 µL of both *Mc*Nrf2 and *Mc*SLC35E2. After injection, the individuals were exposed to Bap separately. Subsequently, the reactive oxygen species (ROS) production and total antioxidant capacity (T-AOC) were detected by using kits (Jian cheng, Nanjing, China).

### 2.8 Statistical analysis

All data were analyzed using SPSS 27.0 software (IBM Corp., Armonk, NY, United States). The results were presented as mean ± SD. Before conducting statistical analysis, normality tests and tests for homogeneity of variances were performed. For comparing two sets of data, the *t*-test was utilized. Data with more than two sets were analyzed using one-way analysis of variance (ANOVA), followed by Tukey’s multiple range test for *post hoc* comparisons. Statistical significance was considered for probabilities of *p* < 0.05.

## 3 Results

### 3.1 ChIP-seq data analysis

**FIGURE 1:**
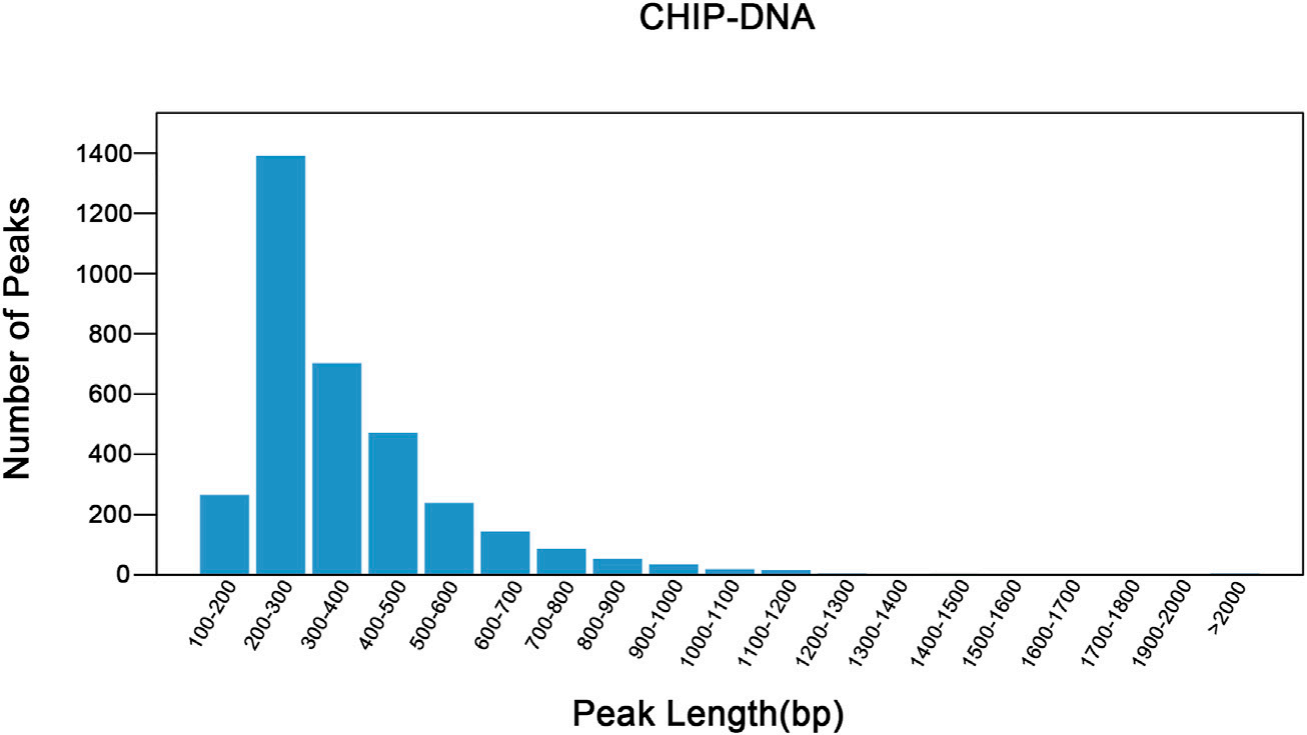
Peak length distribution. The abscissa shows the peak length and the ordinate shows the number of the peaks. The plot illustrates the distribution of peak lengths and provides insights into the frequency of peaks with different lengths in the dataset.

**FIGURE 2:**
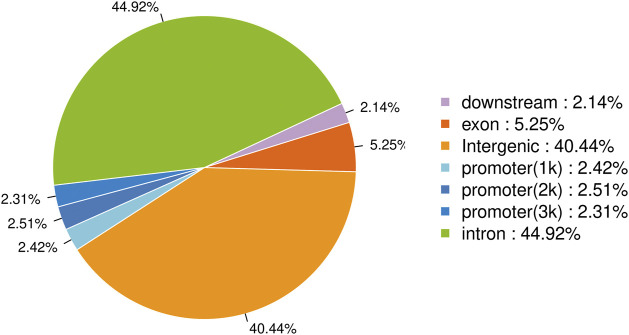
Distribution of peaks in genomic regions.

The raw data has been uploaded to the GEO database with the accession number GSE242277. The raw data from the sample (Nrf2 chip) and control (Nrf2 input) groups were 5.96 billion bp and 5.75 billion bp, respectively. After removing low-quality bases or filtering for valid data (Table 1). The quality of the filtered data is high (Q > 30), and the majority of the data surpasses this threshold, indicating that the reads are of high quality. After quality control, 79.44% of the unique reads were localized to *M. coruscus* genome. The statistical analysis revealed a total of 3,465 peaks, with an average peak length of 383.88 bp and a median peak length of 311 bp. The majority of peak lengths clustered around 200–300 bp (Figure 1). Annotation of 3,465 peaks was conducted to obtain comprehensive information about all the identified peaks in the genome (Supplementary Table S2). Among all the peaks, 7.24% are situated in the promoter transcription start site (TSS) regions (Figure 2). The majority of peaks are located in intergenic regions and introns (Figure 2).

**TABLE 1:** Statistical summary of ChIP-seq raw data after filtration (average >Q30).

Samples	Raw reads	Raw bases	Clean reads	Clean bases	Clean Q30	Clean rate (%)
Nrf2 chip1	21,213,802	3,182,070,300	20,448,090	2,988,027,409	93.22	93.90
Nrf2 chip2	21,213,802	3,182,070,300	20,448,090	2,973,059,420	90.19	93.43
Nrf2 input1	20,892,596	3,133,889,400	20,056,089	2,883,119,713	93.32	92.00
Nrf2 input2	20,892,596	3,133,889,400	20,056,089	2,866,943,649	89.92	91.48

### 3.2 Annotation of genes identified by Nrf2 ChIP

**FIGURE 3:**
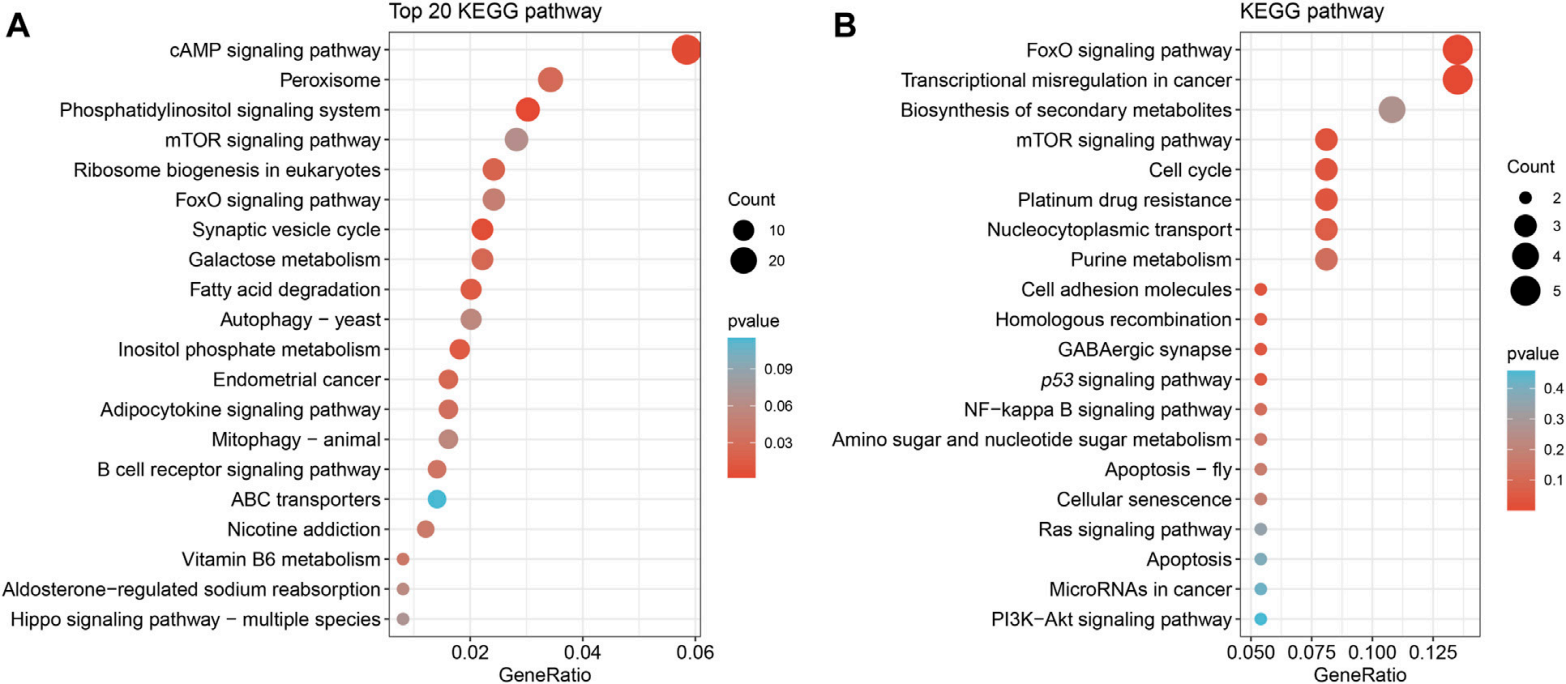
Kyoto Encyclopedia of Genes and Genomes (KEGG) pathway enrichment analysis. **(A)** A total of 3,465 peak KEGG enrichment analyses unveiled signaling pathways. **(B)** The analysis of 219 peak KEGG enrichment, situated within the promoter region, unveiled signaling pathways.

**FIGURE 4:**
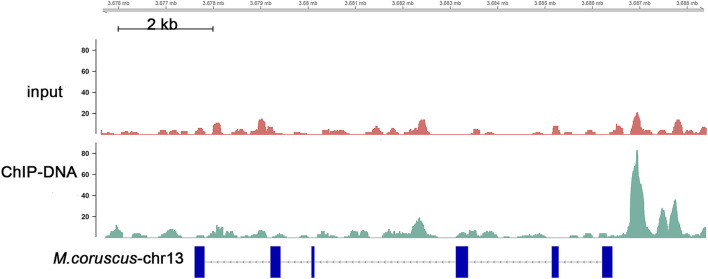
Distribution of SLC35E2 in the genome situation. Each data point or peak on the plot represents the localization of SLC35E2 within the genome. The *x*-axis represents the genomic coordinates, and the *y*-axis shows the intensity or frequency of SLC35E2 at each location.

To obtain a comprehensive set of Nrf2 binding sites, we performed ChIP-seq analysis using the digestive gland of *M. coruscus*. In total, 3,465 peaks were identified as potential binding sites, and among them, 89.24% of the peaks were successfully annotated to the nearest gene. Out of all the peaks, 219 were localized within the promoter zone. Gene functions were established through the utilization of information sourced from diverse databases, notably Swiss-Prot, Interpro, TrEMBL, and KEGG databases. A KEGG enrichment analysis was conducted on a total of 3,465 screened peaks, revealing significant signaling pathways linked to Nrf2 target genes, which included Phosphatidylinositol signaling system (map04070), cAMP signaling pathway (map04024), and Peroxisome (map04146) (Figure 3A). The analysis of enrichment for 219 peaks within the promoter region yielded the subsequent pathways: FoxO signaling pathway (map04068), mTOR signaling pathway (map04150), and p53 signaling pathway (map04115) (Figure 3B). Binding sites located in promoter regions (1K) are likely to be highly regulated by Nrf2. We conducted a screening of our Nrf2 target gene of interest, *SLC35E2*, for which the regulatory relationship with Nrf2 has not been previously mentioned. *SLC35E2* annotated to *M. coruscus* chromosome 13 and there was a clear peak of enrichment for *SLC35E2* compared to the input group (Figure 4).

### 3.3 Nrf2-specific binding sites

Transcription factors’ DNA binding sites often exist as conserved short sequence fragments. Therefore, motif analysis of the ChIP-seq results aids in analyzing the recognition pattern of transcription factors on DNA sequences. Predictions were screened to assess the potential binding of Nrf2 to the identified motif (Table 2).

**TABLE 2:** CentriMo enrichment motif for *de novo* results.

Rank	Motif	E-value
1	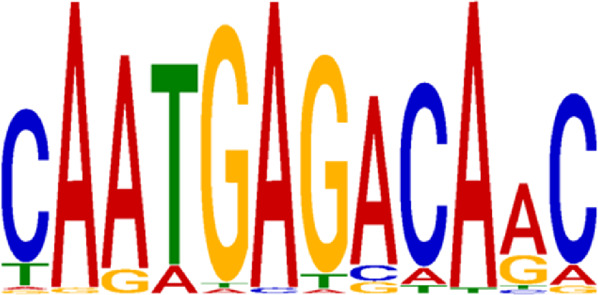	9.30E-03
2	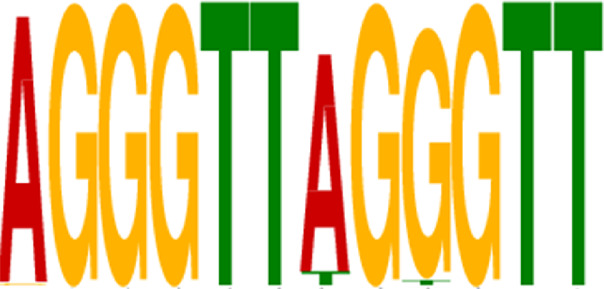	4.20E-55
3	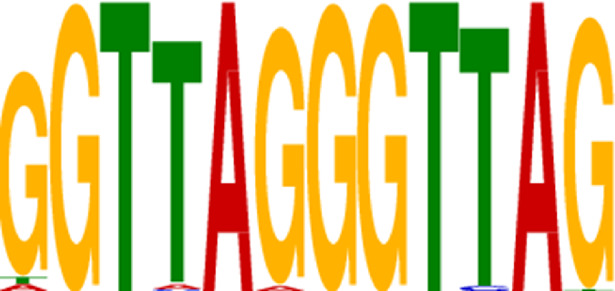	3.30E-61

### 3.4 Expression patterns and regulatory relationships of target genes

**FIGURE 5:**
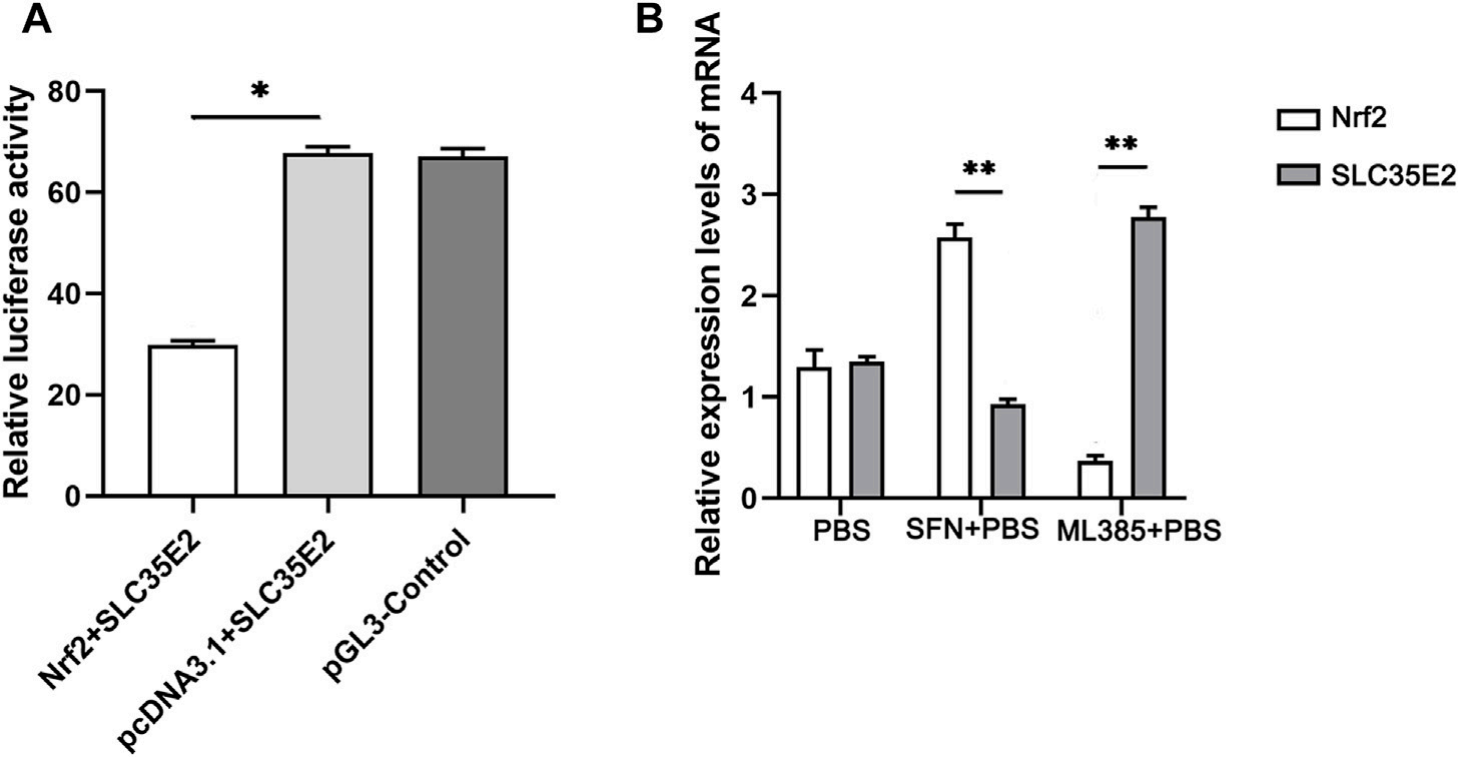
Verification of regulatory relationship between Nrf2 and SLC35E2. **(A)** The relative activity of luciferase. The control group was pGL3-control plasmid. The vertical bars represent the mean ± standard deviation (SD) (*n* = 3); *p < 0.05 **(B)** Expression levels of Nrf2 and SLC35E2 genes after SFN and ML385 treatment. The vertical bars represent the mean ± SD (*n* = 3). **p < 0.01.

To validate the targeting relationship between SLC35E2 and Nrf2, a dual luciferase assay was employed. The activity of Firefly luciferase was divided by the activity of Renilla luciferase to assess the regulatory effect of SLC35E2 and the role of the transcription factor Nrf2 on SLC35E2. The highest luciferase activity was observed for pcDNA3.1+SLC35E2, whereas Nrf2+SLC35E2 luciferase activity was lower (*p* < 0.05) (Figure 5A). This indicates that the presence of Nrf2 leads to a reduction in the expression of SLC35E2. To explore the regulatory relationship between Nrf2 and SLC35E2, we utilized the Nrf2 agonist SFN and the Nrf2 inhibitor ML385. In comparison to the control group, the expression of *Nrf2* increased following SFN treatment, while the expression of *SLC35E2* decreased significantly (*p* < 0.05) (Figure 5B). In contrast, *Nrf2* expression was reduced, and *SLC35E2* expression significantly increased after ML385 treatment (*p* < 0.05) (Figure 5B).

### 3.5 Nrf2 target genes and oxidative stress regulation

**FIGURE 6:**
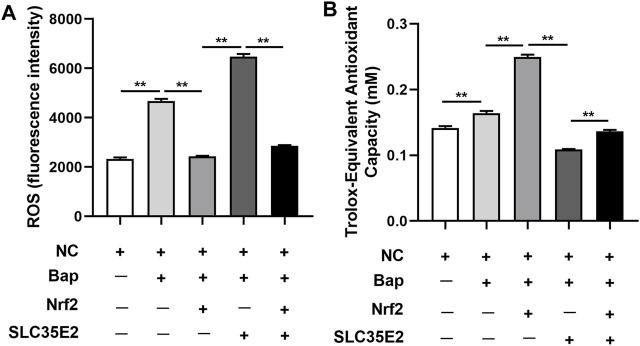
Nrf2 targeting SLC35E2 is involved in the antioxidant effect of Bap exposure. **(A)** ROS levels detected. **(B)** T-AOS levels detected. The vertical bars represent the mean ± SD (*n* = 3). **p < 0.01.

*Mc*Nrf2 and *Mc*SLC35E2 plasmids showed differences in ROS levels after injection and Bap exposure. As depicted in Figure 6A, the level of ROS was higher in the presence of Bap compared to the control group (NC) (*p* < 0.05). However, when Bap was exposed and Nrf2 was overexpressed, the level of ROS was reduced (*p* < 0.05). On the other hand, elevating the level of SLC35E2 was associated with increased ROS levels (*p* < 0.05). Nevertheless, when both Nrf2 and SLC35E2 were overexpressed, the level of ROS was lower than when only SLC35E2 was overexpressed (*p* < 0.05). After injection, there was a notable difference in T-AOC levels in the digestive gland, as depicted in Figure 6B. T-AOC was elevated after exposure to Bap and Nrf2 overexpression in comparison to the NC group (*p* < 0.05). Moreover, T-AOC was decreased in the SLC35E2 overexpression group compared to the Nrf2 overexpression group (*p* < 0.05). However, when both Nrf2 and SLC35E2 were overexpressed, T-AOC levels were elevated compared to SLC35E2 overexpression (*p* < 0.05).

## 4 Discussion

ChIP-seq is an exceptionally powerful technique for identifying specific transcription factor binding sites (Bansal et al., 2015). Its applications have been expanding rapidly, with recent studies successfully implementing this method in different species. Regrettably, the application of this technology to marine mollusks is still in its infancy. Liu et al. (2020) successfully established the ChIP-seq method in *Crassostrea gigas*. To our knowledge, this is the first application of this technology in marine mollusks. In this study, the researchers employed ChIP-seq technique to scan genes regulated by HSF1. The sequencing yielded a set of unique reads, with a 34.2% match rate to the *C. gigas* genome. Ultimately, a total of 916 peaks corresponding to HSF1 binding sites were identified, of which 6% were located in the TSS region, and a subset of HSP genes displayed a direct binding to HSF1. In the present study, unique reads showed a higher genome matching degree (79.44%), indicating high sequencing quality. Statistical analysis revealed a total of 3,465 peaks corresponding to Nrf2 binding sites, 7.24% of which were located at TSS region, and most of the peaks were located at intergenic regions and introns. Our results aligned with the results of a prior investigation, that Nrf2-ChIP-seq data from A549 cells also revealed an approximately 7% gene binding sites on the TSS promoter (Namani et al., 2019). This consistency between our data and the previous study reinforced the reliability and validity of the present findings.

The KEGG analysis of peaks showed a predominant enrichment in the FoxO, mTOR, and p53 signaling pathway. The FoxO signaling pathway has been found to be involved in various aspects, including lifespan regulation, growth and development, as well as resistance to starvation and environmental stressors (Xiao et al., 2018; Wang et al., 2021a; Chen et al., 2023). Considering that Nrf2 acts as a crucial trigger for the body’s antioxidant defense mechanisms, the significant association between *Mc*Nrf2 and FoxO implies that when *M. coruscus* mussels face oxidative stress, the activation of the FoxO pathway regulates growth and development, ultimately ensuring the maintenance of normal life activities. In shellfish, the mTOR signaling pathway also acts as a key player, orchestrating crucial processes such as enhanced lysosomal membrane permeability and the initiation of autophagy (Sforzini et al., 2018). This pathway is constantly vigilant and responds to changing environmental conditions, shaping shellfish cell metabolism and growth strategies accordingly. It is widely believed that invertebrates in marine environments encounter various stressors, including pollutants, low oxygen, and pathogens. Studies have shown that the p53 pathway promotes stress response and cell apoptosis in bivalve cells under various stressors (Xie et al., 2022). Nrf2-targeted genes were highly enriched in the mTOR and p53 pathways, indicating that Nrf2 indeed plays an important role in bivalve’s physiological responses to stressors, which may be associated with immune response, cell cycle regulation, cell apoptosis, and other processes.

Shin et al. (2013) investigated the functional roles of Nrf2 target genes including glutamate cysteine ligase (*GCLC*), *NAD*(*P*)*H*, quinone oxidoreductase 1 (*NQO-1*), UDP-glucuronosyltransferase (*UGT*), and hemeoxygenase-1 (*HO-1*), in hepatic pathophysiology. They found that these genes play complex and multifaceted roles in liver inflammation, fibrosis, and hepatocarcinogenesis. Nrf2 plays a positive role in the equilibrium state, however, the imbalance of Nrf2 and its target gene expression will inflict severe damage upon the organism. Kong et al. (2021) found that sustained high expression of Nrf2 and its target genes, *NQO1* and B-cell lymphoma-2 (*BCL-2*), induced dysplasia of cell proliferation and apoptosis, and were associated with malignant transformation of human bronchial epithelial cells induced by arsenite. Indeed, the most important role of Nrf2 target genes lies in their contribution to the antioxidant defense system and their ability to mitigate oxidative damage. Nrf2 target genes ensure cell integrity and overall health in the face of oxidative challenges by upregulating antioxidant enzymes and detoxifying proteins (Wang et al., 2021b). Unfortunately, studies of Nrf2 and its target genes have been more extensive in humans and mammals, but there has been very limited reporting in aquatic organisms, especially bivalve mollusks.

In the present study, ChIP-seq scanned a total of 219 candidate target genes of *Mc*Nrf2 with binding sites located within the promoter region, and the enriched peaks corresponding to the putative binding sites of *SLC35E2* and Nrf2 were identified by comparing the sequence reads with and without Nrf2 antibody treatment. Laboratory experiments including the dual luciferase and qRT-PCR assays were employed to verify the *in silico* prediction. Dual luciferase assay showed that compared with *Mc*SLC35E2 alone, the luciferase activity in the Nrf2 supplemental group was lower, indicating that *Mc*Nrf2 could target *Mc*SLC35E2 and was negatively correlated. The qRT-PCR further confirmed this fact, that the transcriptional expression of *McSLC35E2* was activated by Nrf2 inhibitor ML385 while inhibited by Nrf2 agonist SFN. These results suggested that *Mc*Nrf2 may be involved in the regulation of physiological processes in *M. coruscus* mussels by inhibiting *Mc*SLC35E2.

Members of the SLC family play a crucial role in human physiology as transporters that facilitate the transportation of hydrophilic compounds into and out of cells and subcellular organelles. For instance, SLC30 and SLC39 transport zinc, SLC11 and SLC40 transport iron, and SLC19 transport folic acid and thiamine (Lin et al., 2015). In addition, several SLC35 transporter proteins, including the SLC35E2 subfamily, are considered orphan SLC35 transporter proteins due to their unclear physiological functions and substrate specificity (Parker and Newstead, 2019). However, recent studies have revealed that these orphan transporters may not be directly involved in glycosylation processes (Li et al., 2022). Similarly, Sosicka et al. (2019) provided support for the notion that the SLC35 protein family may have diverse roles beyond glycosylation. For example, SLC35D3 enhances the formation of protein complexes associated with autophagy (Meng et al., 2012), while SLC35A4 plays a critical role in subcellular distribution (Sosicka et al., 2017). Additionally, SLC35F2 has been found to promote the progression of papillary thyroid carcinoma (He et al., 2018). As for SLC35E2, the oncogenic effect was confirmed *in vivo* using a mouse tumor model (Li et al., 2022). In addition, the scRNA-seq technique has proved that SLC35E2 mutations are associated with human disease variants (Cuomo et al., 2022). In summary, the SLC family plays a multifunctional role in various physiological activities. Regrettably, limited research has been conducted on SLC35E2. In order to investigate the involvement of *Mc*Nrf2 in Bap-induced antioxidant effects through its targeting of *Mc*SLC35E2, enzyme activities were determined in the digestive glands injected with the recombinant plasmids of *Mc*Nrf2 and *Mc*SLC35E2 under or non-Bap.

Bap, being one of the most toxic types of PAHs, has been extensively characterized toxicologically (Bieser et al., 2011). The detoxification process of PAHs can generate numerous active intermediates and ROS substances, which can interfere with the normal physiological functions of shellfish (Liu et al., 2014). At this stage, the Nrf2 pathway is activated, which further triggers the expression of a series of antioxidant enzyme genes, resulting in the increase of T-AOC in the body. This elevation of T-AOC helps to reduce ROS production and oxidative stress (Ma and He, 2012; Cheng et al., 2022). Consistent with this, ROS production and T-AOC levels in the digestive glands of *M. coruscus* exposed to Bap were significantly increased in the present study compared with the NC group, indicating that the Bap burst caused severe oxidative stress to the mussels. Overexpression of *Mc*Nrf2 leads to a significant reduction in ROS production, on the contrary, a significant increase in T-AOC levels is observed. Similar results were found in zebrafish studies, where Shi and Zhou (2010) demonstrated that zebrafish embryos exposed to POPs exhibited elevated ROS production and increased oxidative stress, whereas ROS levels decreased when Nrf2 was upregulated. It was worth noting that when *Mc*SLC35E2 is overexpressed, ROS production is significantly increased and T-AOC is significantly decreased. This result demonstrated that *Mc*SLC35E2 may exacerbate oxidative damage, leading to increased oxidative stress in mussels. However, when *Mc*Nrf2 and *Mc*SLC35E2 were both overexpressed, the situation is exactly the opposite, suggesting that this two had antagonistic effects on the oxidative stress induced by Bap in mussels.

## 5 Conclusion

In this study, ChIP-seq technique was employed to identify new target genes of *Mc*Nrf2 in *M. coruscus*. After comprehensive genome-wide survey, 3,465 candidate target genes of *Mc*Nrf2 were scanned, of which 219 owned binding sites located within the promoter region. Following, a typical target gene termed *McSLC35E2* was selected to perform the experimental verification. Specifically, the targeting of *Mc*Nrf2 to *Mc*SLC35E2 was successfully verified using both dual luciferase and qRT-PCR assay. In order to investigate the involvement of *Mc*Nrf2 in Bap-induced antioxidant effects through its targeting of *Mc*SLC35E2, enzyme activities were determined in the digestive glands injected with the recombinant plasmids of *Mc*Nrf2 and *Mc*SLC35E2 under or non-Bap. The results revealed that *Mc*Nrf2 could participate in the anti-Bap oxidative stress process by inhibiting *Mc*SLC35E2. Overall, these findings lay the groundwork for applying ChIP-seq technology in mollusks, opening up new avenues for understanding the function of Nrf2 in the antioxidant defense system of marine mollusks. The study contributes valuable knowledge that may have implications for future research on environmental responses and stress adaptation in mollusks.

## Data Availability

The original contributions presented in the study are included in the article/Supplementary Material, further inquiries can be directed to the corresponding author.
